# C16 Peptide Promotes Vascular Growth and Reduces Inflammation in a Neuromyelitis Optica Model

**DOI:** 10.3389/fphar.2019.01373

**Published:** 2019-12-03

**Authors:** Haohao Chen, Xiaoxiao Fu, Jinzhan Jiang, Shu Han

**Affiliations:** ^1 ^Medical Molecular Biology Laboratory, School of Medicine, Jinhua Polytechnic, Jinhua, China; ^2^Institute of Anatomy and Cell Biology, Medical College, Zhejiang University, Hangzhou, China

**Keywords:** C16 peptide, receptor tyrosine kinase Tie2, PI3K/Akt pathways, αvβ3 integrin, neuromyelitis optica (NMO)

## Abstract

The goal of this study was to elucidate the mechanism of action of C16, a laminin-1 peptide that competes with αvβ3 for integrin binding, in treating neuromyelitis optica (NMO). A NMO rat model was established and specific inhibitors were used to investigate the effect of Tie2 kinase, integrin, and PI3K/Akt signaling pathways on C16 function in NMO using histological, immunohistochemical, immunofluorescence, Western blot, and ELISA assays. A total of 150 rats were divided into five groups: a control untreated group (*n* = 18) and four test groups (*n* = 33 per group) including vehicle-treated control, C16, Tie2 kinase inhibitor + C16, and PI3K/Akt inhibitor LY294002 + C16. We found that inhibiting Tie2 kinase resulted in partial loss of C16 peptide-mediated effects, while suppressing PI3K/Akt signaling reduced C16 peptide-mediated effects. In addition, activation of the αvβ3 integrin axis and Tie2 kinase promoted PI3K/Akt signaling. Our study showed that the Tie2-PI3K/Akt, Tie2 integrin, and integrin-PI3K/Akt signaling pathways regulate C16 peptide function in vascular growth and stabilization as well as inflammation in NMO.

## Introduction

Disruption of the blood–brain barrier (BBB), which plays a crucial role in maintaining cerebral homeostasis, is the rate-determining step in the progression of inflammatory neurological diseases, such as multiple sclerosis (MS) and neuromyelitis optica (NMO) (Shimizu and Kanda, 2013). NMO is an autoimmune inflammatory demyelinating disease that mainly affects the spinal cord (SC) and optic nerve (ON), thereby leading to paralysis and blindness, respectively. Characteristic pathological changes in NMO include loss of blood–SC barrier integrity, blood vessel leakiness, and inflammatory cell infiltration (Jiang et al., 2014). Extravasated inflammatory cells can activate factors that can destroy the central nervous system (CNS) microenvironment, making it vulnerable to demyelination and axonal damage, thereby leading to secondary injury and loss of function.

Injection of sera from NMO patients reduced the expression of tight junction proteins, thereby disrupting the BBB (Shimizu et al., 2012). In addition, circulating inflammatory cytokines such as tumor necrosis factor-α (TNF-α), interleukin (IL)-1, and IL-8 have been implicated in BBB breakdown (Shimizu et al., 2012; Tasaki et al., 2014). The increase in blood vessel permeability promotes NMO-IgG antibody invasion of the parenchyma tissues in the CNS, wherein the circulating anti-aquaporin 4 (AQP4) NMO-IgG binds selectively to AQP4 water channels localized at astrocytic foot processes at the BBB. This leads to AQP4 loss, further destruction of the BBB integrity, and NMO progression (Shimizu et al., 2012). Anti-inflammation therapy can protect the motor neurons and alleviate clinical motor symptoms (Jiang et al., 2014). Repairing the integrity of the blood vessels and promoting angiogenesis are novel approaches proposed in NMO therapy that could potentially reduce AQP4-IgG-mediated astrocytic damage and AQP4 loss (Shimizu and Kanda, 2013).

The integrin family of proteins participates in a variety of physiological and pathological events including cell adhesion, cell migration, intracellular signaling pathway activation, and cytoskeletal formation (McCarty et al., 2005). As an integral part of the vascular endothelium, integrins also mediate the transmigration of monocytes into inflamed tissues, thus contributing to the initiation and progression of inflammation (Patarroyo, 1994). Cells in the CNS microenvironment release factors such as αv integrins that influence blood vessel growth and survival. The αv integrins play an important role in mediating the interactions between embryonic cerebral blood vessels and brain parenchymal cells. Hence, αv integrin deletion in CNS glia and neurons causes cerebral hemorrhage (McCarty et al., 2005).

The C16 peptide, a γ1 chain peptide of laminin-1, was reported to promote angiogenesis and reduce blood vessel leakiness by binding with its agonists, αvβ3 and α5β1 integrins. This effect could further competitively block monocyte adhesion and transmigration through the vascular endothelium, which is initially mediated by monocytes adhering to αvβ3/α5β1 integrin on the surface of vascular endothelial cells (Patarroyo, 1994; Weerasinghe et al., 1998; Ponce et al., 2001; Ponce and Kleinman, 2003; Han et al., 2010).

Natalizumab was shown in a case study by Kleiter et al. to be ineffective at treating NMO patients, but was effective for treating relapsing-remitting MS (Kleiter et al., 2012). Natalizumab can reportedly reduce the adhesion of immune cells to the BBB by blocking the α4 integrin on the surface of immune cells. However, natalizumab only functions as an antagonist of α4 integrin, while C16 is an agonist for both ανβ3 and α5β1. The different roles of C16 suggest that it likely has a different mechanism of action from natalizumab and that it may be effective in NMO therapy (Ponce et al., 2001).

Since the PI3K/Akt pathway has a well-known role in angiogenesis (DeBusk et al., 2004), we hypothesized that binding of the C16 peptide to integrins activates the endothelial receptor tyrosine kinase Tie2 (Tie2 KI) and PI3K/Akt pathways to prevent NMO progression. Angiotensin-1 (Ang-1), which is a Tie2 KI agonist (Maisonpierre et al., 1997; Brindle et al., 2006; Makinde and Agrawal, 2008), induces Tie2 translocation to cell–cell junctions (Thomas et al., 2010), and in turn induces Tie2-dependent Akt activation and subsequent survival signaling (Brindle et al., 2006). The crosstalk between Tie2 and integrins (αvβ1 and α5β3 integrins) regulates Tie2 activation and signaling which enables endothelial cell (EC) survival and motility (Cascone et al., 2005; Shlamkovich et al., 2018). Hence, in this study, we used specific inhibitors of Tie2 KI and PI3K to investigate the downstream effects of C16 peptide in an NMO rat model. Specifically, we determined the effects of C16 on the BBB, blood vessel leakage, and infiltration of inflammatory cells.

## Materials and Methods

### Animals and Grouping

A total of 150 adult male Lewis rats weighing 250–300 g were obtained from the Laboratory Animal Services Centre of Zhejiang University, China. Eighteen rats were included in the untreated control group, and 132 rats were included in the NMO group.

The NMO rats were randomly divided into four groups: the vehicle-treated group (*n* = 33), wherein the rats were intravenously injected with 1 ml of phosphate-buffered saline (PBS) daily for 2 weeks; the C16-treated group (*n* = 33), wherein the rats were intravenously injected with 2 mg of C16 peptide (Shanghai Science Peptide Biological Technology Co., Ltd., Shanghai, China) daily for 2 weeks; the C16 and Tie2 kinase inhibitor-treated group (Tie2 KI + C16 group; *n* = 33), wherein the rats were intravenously injected with 2 mg of C16 peptide daily for 2 weeks and intraperitoneally injected with 25 mg/kg of the Tie2 kinase inhibitor (Selleck, Shanghai, China) daily for 2 weeks; and the C16 peptide and LY294002-treated group (LY294002 + C16 group; *n* = 33), wherein the rats were intravenously injected with 2 mg of C16 peptide daily for 2 weeks and intraperitoneally injected with 100 mg/kg of the class I PI3K inhibitor LY294002 (Selleck, Shanghai, China) daily for 2 weeks.

### Induction of the NMO Rat Model

We obtained serum from two patients from Sir Run Run Shaw Hospital (SRRSH) who had an established diagnosis of NMO and strong AQP4 autoantibody serum positivity. AQP4-Ab was purified as described previously (Gruneward et al., 2016) and its titers were independently measured using fluoroimmunoprecipitation and cell-based assays. To induce NMO in the male Lewis rats, the rats were first anesthetized with 1% nembutal (40 mg/kg, i.p.) before injection of AQP4-Ab. The coordinates of the intraventricular injections performed were as follows: anteroposterior (AP), −0.7 mm; mediolateral (ML), −1.7 mm from the bregma; and depth, 5 mm from the skull surface. For continuous administration of AQP4-Ab, an osmotic minipump (Alzet 1003D, Cupertino, CA, USA) delivered 3.3 µg AQP4-Ab and 16.7 µl human complement per day for 3 days (1 µl/h). The vertebrae were carefully separated to expose the lumbar spinal cord (L4–L5) and the same amount of NMO-IgG and human complement was infused for 3 days intrathecally also by similar Alzet 1003D minipumps and catheters (Asavapanumas et al., 2014). Using this method, we successfully created the NMO model. The AQP4-Ab serum levels in this rat model were 1.36:1 (mg/ml, *P* < 0.05) relative to the normal rats (data not shown). All animal procedures performed in this study were carried out in accordance with the US National Institute of Health Guide for the Care and Use of Laboratory Animals. This study was approved by the animal ethics committee of Zhejiang University, China.

### Animal Scoring

Disease severity of treated rats was assessed daily as previously described (Gruneward et al., 2016) using a 0 to 10 scale: 0, normal; 1, reduced tone of the tail; 2, limp tail, impaired righting; 3, absent righting; 4, gait ataxia; 5, mild paraparesis of the hindlimb; 6, moderate paraparesis; 7, severe paraparesis or paraplegia; 8, tetraparesis; 9, moribund; and 10, death.

### Perfusion and Tissue Processing

Animals in the vehicle control and C16-treated groups were sacrificed post-immunization (P.I.) at 3 and 8 weeks (five rats per time point per group). Rats were anesthetized with sodium pentobarbital and perfused intracardially with cold saline followed by 4% paraformaldehyde in 0.1 M phosphate buffer (pH 7.4) before carefully harvesting and dissecting the SC and eyeballs. The lumbar SC (1 cm) and an eyeball of each rat were fixed in 4% paraformaldehyde for 4 h and then soaked in a solution of 30% sucrose in PBS until the tissues sank to the bottom of the container. A freezing microtome and a Leica cryostat (Buffalo Grove, IL, USA) were used to obtain 20-µm-thick brain and SC sections, respectively. These sections were then mounted onto 0.02% poly-l-lysine-coated slides for histological, immunohistological, and immunofluorescent staining.

### Transmission Electron Microscopy

The remaining CNS tissues (different regions of white matter) and another eyeball were fixed in 2.5% glutaraldehyde solution and then washed three times with 0.1 M PBS before being immersed in 1% osmium tetroxide at 4°C overnight. The sections were then washed three times with 0.1 M PBS before examination using a transmission electron microscope (TEM, JEOL Ltd. Tokyo, Japan) as previously described (Wang et al., 2016).

To detect endothelial cell survival in perfused blood vessels, the jugular vein was intravenously injected with 100 mg/100 ml fluorescein isothiocyanate (FITC)-conjugated *Lycopersicon esculentum* (tomato) agglutinin lectin (LEA, which labels perfused vasculature; Patarroyo, 1994) for 30 min before euthanasia was performed. The number of LEA-labeled vessels that intersected 100-mm-spaced horizontal (five) and vertical (six) lines were counted in each image and the volume per section interval was calculated using the NIH image processing software (NIH, Bethesda, MD, USA).

### Histological Assessment by Nissl and H&E Staining

Neurons with a well-defined nucleolus, cell body, and high density of endoplasmic reticulum from both anterior horns of the spinal cord were stained with cresyl violet (Nissl staining) and visualized under a Nikon TE-300 microscope (Nikon, Japan) at ×200 bright-field magnification to assess the degree of inflammation and neuronal survival. Three randomly selected fields of view per section were used for counting. The severity of inflammatory cell infiltration was assessed by conventional hematoxylin and eosin (H&E) staining (Ma et al., 2010) and was scored as follows: 0, no inflammation; 1, cellular infiltrates around blood vessels and meninges only; 2, mild cellular infiltrates in parenchyma tissue (1–10/section); 3, moderate cellular infiltrates in parenchyma tissue (11–100/section); and 4, severe cellular infiltrates in parenchyma tissue (100/section).

### Evans Blue Staining to Assess Vascular Permeability

Vascular permeability of the rat BBB (*n* = 3 per group were randomly selected) was assessed using the Evans Blue (EB) extravasation method with modifications (Wang et al., 2016). Briefly, rats at 2 and 8 weeks P.I. were anesthetized with sodium pentobarbital (60 mg/kg, i.p.) and were infused *via* the right femoral vein over 5 min with EB dye (2% in 0.9% normal saline, 4 ml/kg) at 37°C. After 2 h, the blood vessels were perfused with 300 ml normal saline to wash out the remaining dye. The EB-stained 20-µm-thick tissue sections were visualized *via* red laser excitation under an ultraviolet light filter. Tissues with high vascular permeability stained red. The staining intensity was assessed using ImageJ software (NIH, Bethesda, MD, USA).

### Immunohistochemical Staining

Immunohistochemical (IHC) staining was performed as previously described (Weerasinghe et al., 1998; Ponce et al., 2001; Ponce and Kleinman, 2003; Han et al., 2010). A ring of wax was applied around the fixed tissue sections with a water-repellent *pap pen* (Invitrogen, Carlsbad, CA, USA) before rinsing with 0.01 M Tris-buffered saline (TBS) for 10 min. Permeabilization and blocking of the sections were subsequently performed with 0.3% Triton X-100/10% normal goat serum in 0.01 M PBS for 30 min before incubating with rabbit polyclonal anti-CD45 (1:200; Abcam, Cambridge, MA, USA), primary antibodies overnight at 4°C. Sections were incubated with secondary biotinylated goat anti-rabbit IgG antibody (1:400; Vector Laboratories, CA, USA) for 1 h at room temperature, followed by an avidin–biotin peroxidase complex (ABC kit, Thermo Fisher Scientific, CA, USA). After incubation for 5 min with 0.02% DAB and 0.003% H_2_O_2_ in 0.005 Tris-HCl, the sections were counterstained with hematoxylin. Primary antibody controls were used to confirm IHC labeling specificity. Five sections (three visual fields per section) of the motor cortex and anterior horns of the SC of each animal were randomly selected for counting. The stained sections were imaged, and the total number of cells and the number of positive cells were counted in six randomly selected fields under ×200 magnification in triplicates.

### Immunofluorescence Staining

The sections were pretreated with the same method as described for IHC staining before incubating with primary antibodies against polyclonal rabbit anti-glial fibrillary acidic protein (GFAP) (1:1,000; Thermo Fisher Scientific, Waltham, *MA, USA*), anti-AKT (1:500; R&D Systems, Minneapolis, MN, USA), anti-Tie2 (1:500; R&D Systems, Minneapolis, MN, USA), anti-AQP4 (1:500; Santa Cruz Biotechnology, Santa Cruz, CA, USA), anti-αvβ3 and α5β1 integrins (1:1,000; Neuromics, MN, USA), anti-caspase 3 (1:500; Cayman Chemical, Ann Arbor, MI, USA), and mouse anti-myelin basic protein (MBP, 1:1,000; Abcam, Cambridge, MA, USA) at 4°C overnight. Sections were rinsed with PBS three times followed by incubation with FITC/TRITC-conjugated secondary goat anti-rabbit/mouse IgG antibodies (1:200; Invitrogen, Carlsbad, CA, USA) for 1 h at 37°C before mounting with Antifade Gel/Mount aqueous mounting media (Southern Biotech, USA). Control sections were incubated in PBS instead of primary antibody solutions. Immunoreactive areas were analyzed using the NIH image processing software (NIH, Bethesda, MD, USA).

Demyelination and axonal loss were assessed using NF-200 and MBP double staining and were scored using a six-point scale system as follows: 0, normal white matter; 1, rare foci; 2, a few areas of demyelination; 3, confluent perivascular or subpial demyelination; 4, massive perivascular and subpial demyelination involving one half of the SC with the presence of cellular infiltration in the CNS parenchyma; and 5, extensive perivascular and subpial demyelination involving the whole cord section with the presence of cellular infiltration in the CNS parenchyma (Yin et al., 2010). Axonal loss was estimated using the four-point scale (Ma et al., 2010): 0, no axonal loss; 1, a few foci of superficial axonal loss (<25% of the tissues); 2, foci of severe axonal loss (>25% of the tissues); and 3, diffuse and widespread axonal loss (>50% of the tissues). Five transverse sections (three visual fields per section) from each animal at ×200 magnification were randomly selected and imaged using a fluorescence microscope (Leica Microsystems Inc., Buffalo Grove, IL, USA). Histological results were scored by investigators who had no prior knowledge of the treatment groups.

### Counting of Retinal Ganglion Cells

Retinal ganglion cells (RGCs) were counted as previously described (Maisonpierre et al., 1997). Briefly, the brain surface was exposed by drilling the parietal bone using a stereotactic apparatus. Of 5% FluoroGold (Fluorochrome, Denver, CO, USA), 2.1 ml was then injected bilaterally at 5.5 mm caudal to the bregma, 1.2 mm lateral to the midline, and 4.5 mm in depth from the skull surface into the rat’s superior colliculus 5 days before treating with NMO-IgG and human complement (*n* = 3 per group). Isolated RGCs were fixed in 4% paraformaldehyde and then mounted on a glass slide with the RGC layers facing upward. FluoroGold-positive RGCs were identified under a fluorescence microscope using an ultraviolet light filter (377/407 nm). RGCs were counted in groups of 12 (0.072 mm^2^ each; three areas per retinal quadrant) by an investigator with no prior knowledge of the treatment conditions.

### Flow Cytometry Analysis of Spinal Cord Tissue

After isolation of the lumbar spinal cord, single cell suspensions were prepared. The cells were permeabilized with 0.2% Triton-X100 for 20 min, blocked with 3% BSA in PBS, and stained with CD45 antibody (Abcam, Cambridge, MA, USA) for 1 h at room temperature. Subsequently, the suspensions were incubated with appropriate fluorescence goat anti-mouse Alexa-Fluor-488-tagged secondary antibodies (Thermo Fisher Scientific, Waltham, *MA, USA*) for 1 h at room temperature. Excess unbound antibodies were rinsed off with PBS buffer. Flow cytometry was performed using a FACSAria III cytometer (Becton Dickinson Biosciences, USA) equipped with a 488-nm argon laser and the FACSDiva 6.0 software was used for data analysis. An average of 50 × 10^3^ events was analyzed.

### Cytokine Quantification Using Enzyme-Linked Immunosorbent Assay

Peripheral blood samples were collected from rats sacrificed by decapitation at 3 and 8 weeks P.I. (*n* = 5 per time point per group) at 4°C using heparin as an anticoagulant. Within 30 min of collection, the samples were centrifuged at 1,000 × g for 20 min and then at 10,000 × g for 10 min at 4°C for complete platelet removal before storage at −80°C. To assess cytokine expression, plasma samples were incubated for 1 h at 37°C in 96-well plates that were precoated with IL-10, IL-1β, and IL-8 (BioLegend Inc. San Diego, CA) and TNF-α (R&D Systems, Minneapolis, MN, USA) primary antibodies. Next, the samples were incubated with horseradish peroxidase (HRP)-conjugated goat anti-rabbit IgG secondary antibody (1:2,000; Bio-Rad, CA, USA) for 1 h at 37°C. The optical density (OD) of bound proteins was measured at 450 nm using a model 680 microplate reader (Bio-Rad Laboratories, Corston, UK) and the results were analyzed using the GraphPad Prism version 4 software (GraphPad Prism Software, Inc., CA, USA).

### Western Blot Analysis

The rats were sacrificed by decapitation at 2 and 8 weeks P.I. (*n* = 3 per time point per group). The whole brain cortex and a 10-mm lumbar SC segment from each rat were harvested for Western blot analysis. Proteins were separated by 12% SDS-PAGE electrophoresis and were blotted onto a polyvinylidene difluoride membrane. The membrane was subsequently incubated with rabbit polyclonal anti-GFAP (1:200; Thermo Fisher Scientific, Waltham, *MA, USA*), anti-AKT (1:500; R&D Systems, Minneapolis, MN, USA), anti-Tie2 (1:500; R&D Systems, Minneapolis, MN, USA), anti-AQP4 (1:500; Santa Cruz Biotechnology, Santa Cruz, CA, USA), anti-Akt and pAKT (1:500; R&D Systems, Minneapolis, MN, USA), anti-Tie2 (1:2000; R&D Systems, Minneapolis, MN, USA), anti-αvβ3 and α5β1 integrins (1:1000; Neuromics, MN, USA), anti-caspase 3 (1:500; Cayman Chemical, Ann Arbor, MI, USA), and mouse anti-MBP (1:1000; Abcam, Cambridge, MA, USA) primary antibodies for 12 h at room temperature. To normalize protein bands to a gel loading control, the membranes were re-probed with rabbit anti-β-actin antibody (1:5,000; Abcam, MA, USA). The membranes were then incubated with HRP-conjugated goat anti-rabbit secondary antibody (1:5,000; Santa Cruz, CA, USA) and the blots were detected using enhanced chemiluminescence reagent. The incubation step with the primary antibody was omitted for the negative control.

### Statistical Analysis

Statistical analysis was performed using SPSS 13.0 software (Chicago, IL, USA) and graphs were created using GraphPad Prism version 4.0. Kruskal–Wallis nonparametric one-way analysis of variance (ANOVA) followed by *post hoc* analysis to analyze data presented as percentages. Data are presented as the mean ± standard deviation (SD). A value of *P* < 0.05 was considered statistically significant.

## Results

### C16 Delays NMO Progression in Rats

**Figure 1:**
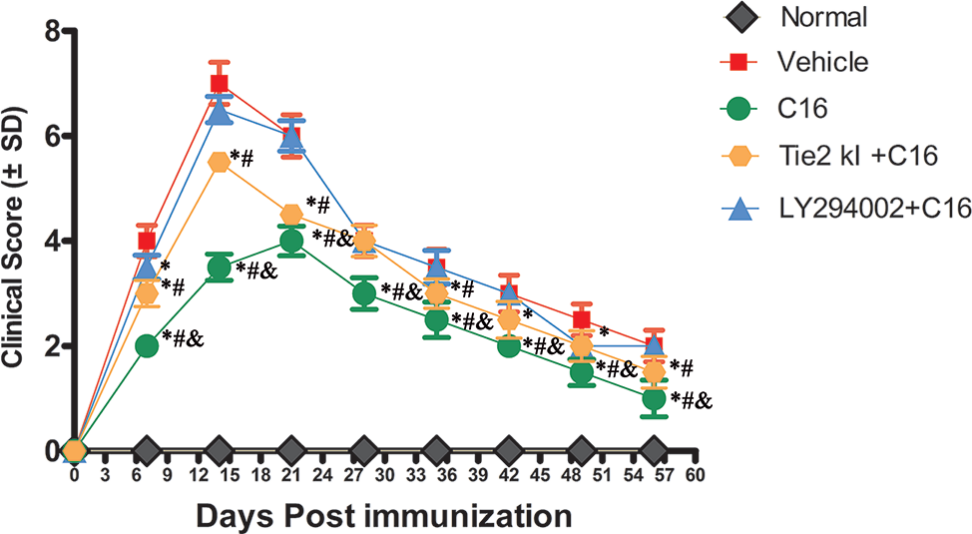
Plot of the neuromyelitis optica (NMO) clinical progression in normal and NMO rats (*n* = 10 in each group). *P < 0.05 versus the vehicle-treated group at the same time point; ^#^ *P* < 0.05 versus the C16 peptide + LY294002-treated group at the same time point; ^&^ *P* < 0.05 versus the Tie-2 KI + C16 group at the same time point.

NMO symptoms started manifesting at 3 days and peaked at 2 weeks P.I. (clinical score of 7 ± 0.4), and the clinical scores remained poor at 8 weeks P.I. in the vehicle control-treated group (**Figure 1**). A limp tail was still observed at 8 weeks P.I. A similar trend was observed in the Tie2 KI + C16 and the C16 peptide + LY294002-treated groups, but with lower clinical scores of 5.5 ± 0.2 and 6.5 ± 0.25 at 2 weeks P.I., respectively (**Figure 1**). In contrast, rats in the C16-treated group showed a delayed peak stage and a significantly reduced severity (clinical score of 4 ± 0.28) at 3 weeks P.I. with gait ataxia. We observed significantly lower clinical scores in the C16-treated group throughout the disease course compared to the other treated groups (*P* < 0 05; **Figure 1**). Similar clinical scores were obtained for the C16 peptide + LY294002-treated group and the vehicle control-treated group at all time points except at 1 week P.I. (*P* > 0.05). The Tie2 KI + C16 group showed lower clinical scores at all time points, except at 4 weeks P.I., compared to the vehicle control-treated group and at all time points, except at 4 and 6 weeks P.I., compared to the C16 peptide + LY294002-treated group (*P* < 0.05; **Figure 1**).

### C16 Reduces Parenchymal Infiltration of Inflammatory Cells and Neuronal Loss and Downregulates Caspase-3 Expression in the CNS of NMO Rats

**Figure 2:**
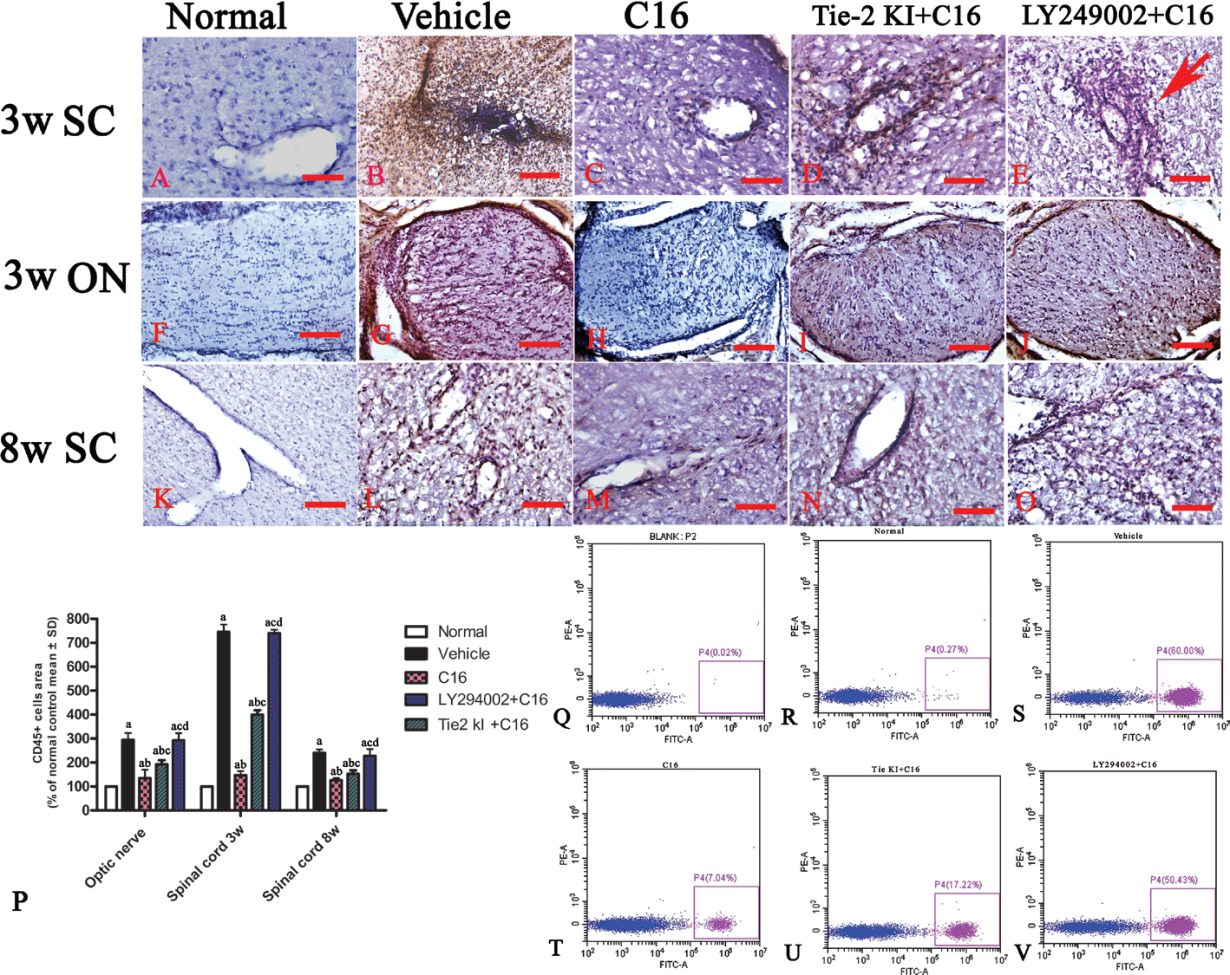
CD45 immunostaining and counterstaining with hematoxylin of transverse sections through the lumbar spinal cord (SC) and optic nerve (ON). *Scale bar*, 100 μm. **(P)** Plot of CD45^+^ leukocytes. a, *P* < 0.05 versus normal rats; b, *P* < 0.05 versus vehicle-treated group; c, *P* < 0.05 versus the C16-treated group; d, *P* < 0.05 versus Tie2 KI + C16 group. **(Q–V)** Flow cytometry of CD45 in the blank, normal, and vehicle control groups, C16, Tie KI + C16, and LY294002 + C16 groups. **(A–O)**: the Infiltration of CD45 + leukocytes in 3 weeks pi spinal cord **(A–E)**, 3 weeks pi optic nerve **(F–J)** and 8 weeks pi spinal cord **(K–O)**.

**Figure 3:**
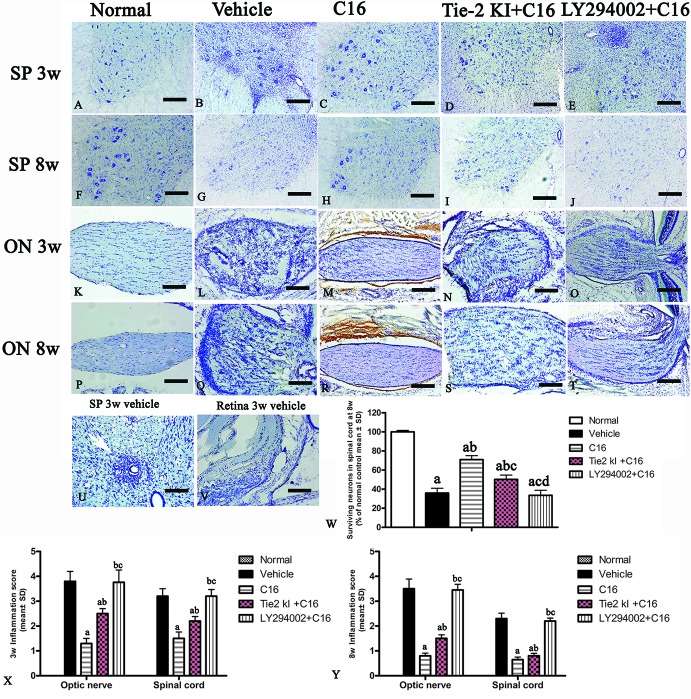
Nissl staining of the transverse sections through the **(A–J, U)** lumbar spinal cord (SC), **(K–T)** optic nerve (ON), and **(V)** retina showing the degree of infiltration of inflammatory cells into the parenchyma tissues of normal and neuromyelitis optica (NMO) rats. *Scale bar*, 100 μm. **(W)** Plot showing the percentage of surviving neurons at 8 weeks post-immunization (P.I.) **(X, Y)** Plots showing the inflammation scoring of each group at 3 and 8 weeks P.I. a, *P* < 0.05 versus normal group; b, *P* < 0.05 versus the vehicle-treated group; c, *P* < 0.05 versus the C16-treated group; d, *P* < 0.05 versus Tie2 KI + C16 group.

Infiltration of inflammatory cells was observed in the parenchyma of the SC, ON, and retina in vehicle-treated NMO rats (**Figures 2** and **3**). A similar result was observed in NMO rats in the C16 peptide + LY294002-treated group (**Figure 2**). Reduced inflammation was observed for the Tie2 KI + C16 group at both 3 and 8 weeks P.I. compared to the vehicle-treated and C16 peptide + LY294002-treated groups (**Figures 2** and **3**). The C16-treated group showed approximately two to three times lower inflammation compared to the Tie2 KI + C16 and C16 peptide + LY294002-treated groups (**Figure 3**). In addition, “perivascular cuffing,” which describes infiltrated leukocytes surrounding blood vessels (**Figures 2B, D, E** and **3U**), was observed in the vehicle-treated, Tie2 KI + C16, and C16 peptide + LY294002-treated groups, but was significantly reduced in the C16-treated group (**Figure 2**). Moreover, the percentage of CD45-labeled cells with respect to total cells was 0.02% in the blank control, 0.27% in the normal control, 60% in the vehicle group, 7.04% in the C16 group, 17.22% in the Tie KI + C16 group, and 50.43% in the LY294002 + C16 group. Thus, the CD45 flow cytometry results confirmed the immunohistochemistry results (**Figure 2**). Neuronal loss from the anterior horn of the SC at 8 weeks P.I. in vehicle-treated NMO rats (**Figure 3G**) could be prevented by C16 treatment (**Figure 3H**) and Tie2 KI + C16 treatment (**Figure 3I**), albeit to a lower degree for the latter, but could not be prevented by LY294002 + C16 treatment (**Figure 3J**).

**Figure 4:**
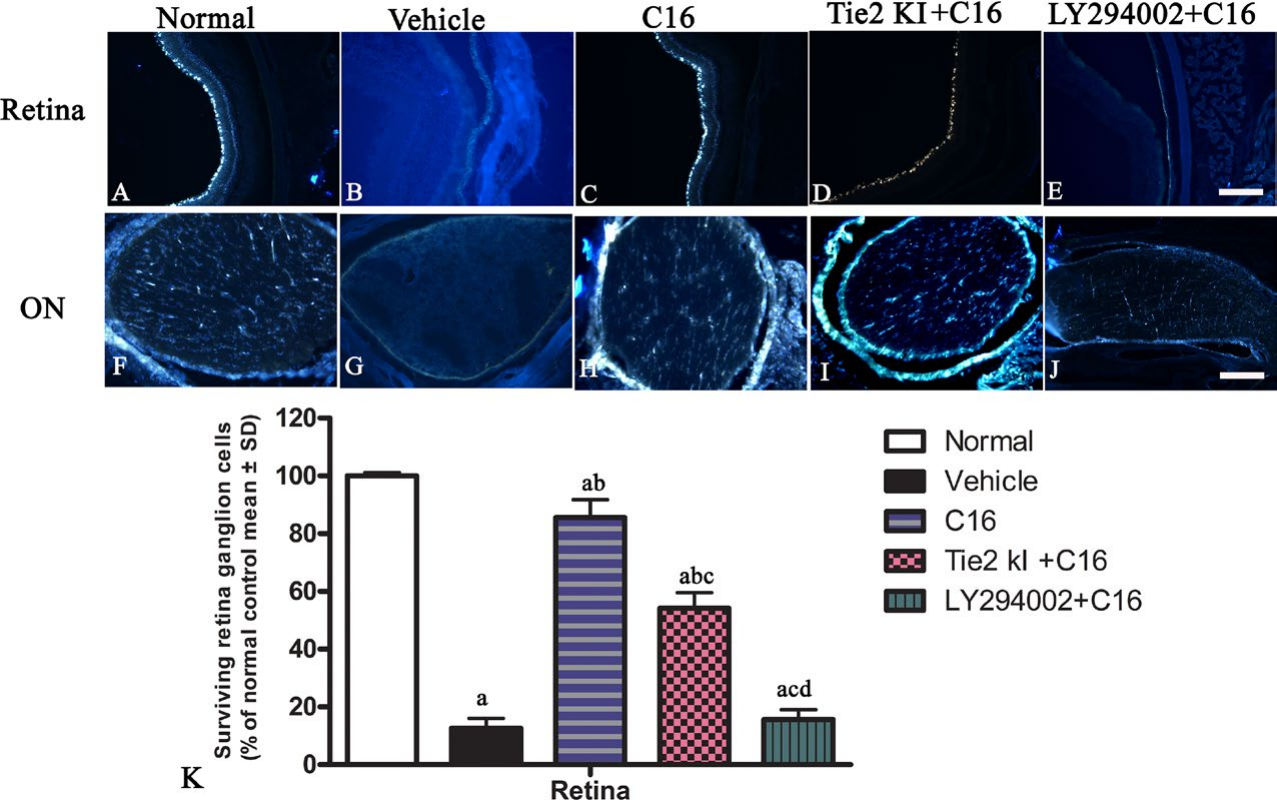
Coronal sections through the retina **(A–E)** and eyeball **(F**–**J)** showing FluoroGold-labeled retinal ganglion cells (RGCs). *Scale*, 100 μm. **(K)** The percentage of surviving RGCs in the five groups (% of control group). a, *P* < 0.05 versus normal group; b, *P* < 0.05 versus the vehicle-treated group; c, *P* < 0 05 versus the C16-treated group; d, *P* < 0.05 versus Tie-2 KI + C16 group.

FluoroGold labeling revealed a significant reduction in the number of surviving RGCs from 100% (normal) to approximately 10% due to NMO-IgG infusion in the vehicle-treated group, and this was similarly observed in the C16 peptide + LY294002-treated group (**Figure 4**). In contrast, C16 (10% RGC loss) and Tie2 KI + C16 (50% RGC loss) treatment prevented RGC loss.

**Figure 5:**
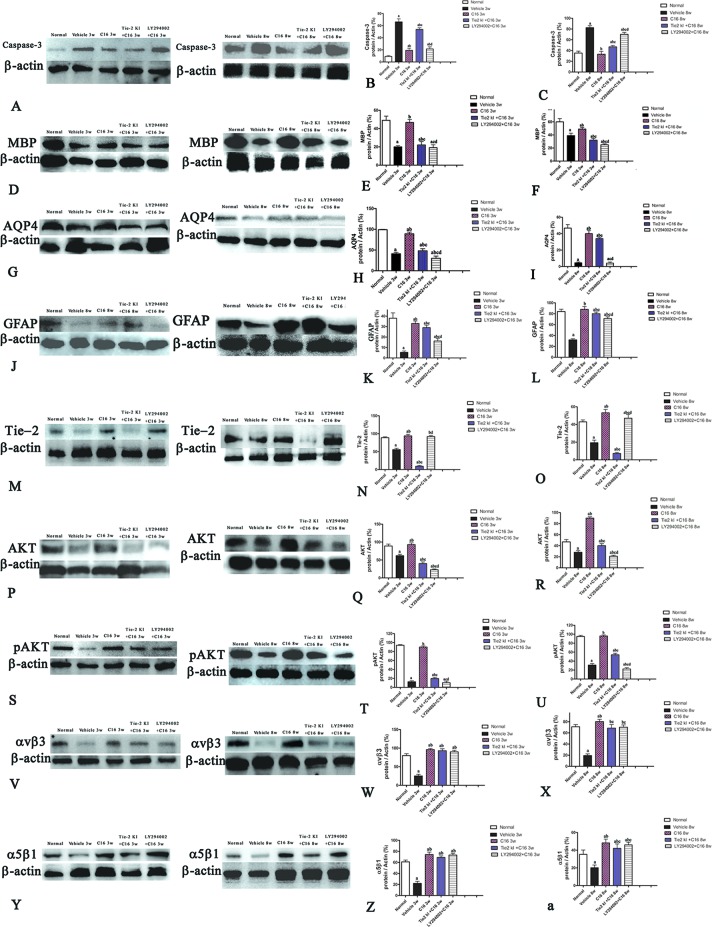
Western blot analysis showing **(A–C)** caspase-3, **(D–F)** myelin basic protein (MBP), **(G–I)** aquaporin 4 (AQP4), **(J–L)** glial fibrillary acidic protein (GFAP), **(M–O)** Tie-2, **(P–R)** Akt, **(S–U)** p-AKT, **(V–X)** αvβ3 integrin, and **(Y-a)** α5β1 integrin expressions in neuromyelitis optica (NMO) rats compared to the normal control group at 3 and 8 weeks post-immunization (P.I.) a, *P* < 0.05 versus normal group; b, *P* < 0.05 versus the vehicle-treated group; c, *P* < 0.05 versus the C16-treated group; d, *P* < 0.05 versus Tie2 KI + C16 group. Panel **(Z)** showed that Inhibition of Tie2 and Akt/pAkt by Tie2 and LY294002-specific inhibitors.

Western blot analysis showed increased expression of the apoptotic marker caspase-3 in NMO rats compared to the normal rats (**Figures 5A–C**). Caspase-3 expression was reduced by treatment with C16, Tie2 KI + C16, or LY294002 + C16 at both 3 and 8 weeks P.I. The C16-treated group showed the lowest caspase-3 expression. Treatment with either Tie2 KI + C16 or LY294002 + C16 showed similarly decreased caspase-3 expression at 3 weeks P.I., but the latter group showed the least effect in preventing caspase-3 expression at 8 weeks P.I.

### C16 and Tie2 KI + C16 Treatment Prevent MBP Downregulation and Reduce Demyelination and Axonal Loss in the CNS of NMO Rats

**Figure 6:**
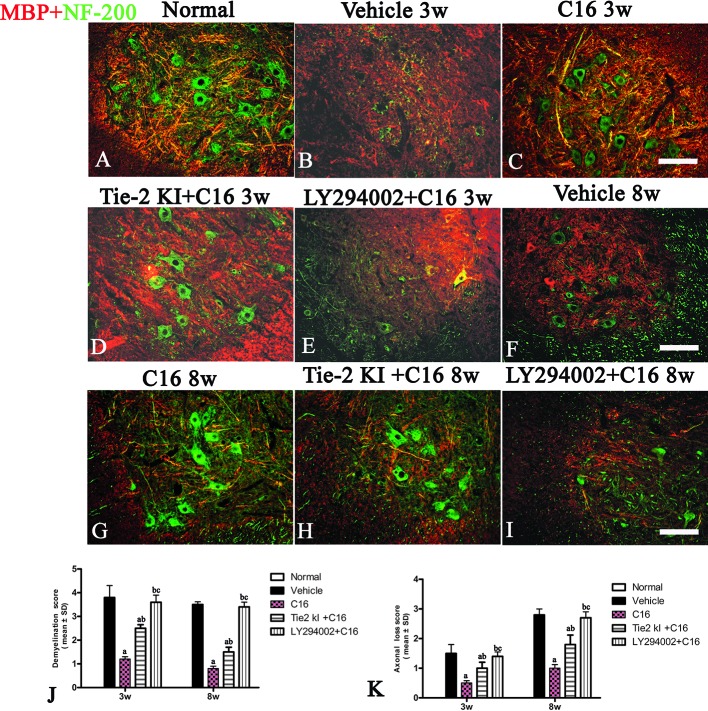
Double immunofluorescence staining images of the transverse sections through the anterior horn of the lumbar spinal cord (SC) showing the distribution of myelin basic protein (MBP, *green*) and neurofilaments (*red*) in **(A)** normal and neuromyelitis optica (NMO) rats of the **(B, F)** vehicle-treated group, **(C, G)** C16 group, **(D, H)** Tie2 KI + C16 group, and **(E, I)** C16 peptide and LY294002-treated group at 3 and 8 weeks post-immunization (P.I.) (MBP, *red*; NF-200, *green*). *Scale bar*, 100 μm. Plots of **(J)** demyelination scoring and **(K)** axonal scoring. a, *P* < 0.05 versus the vehicle-treated group; b, *P* < 0.05 versus the C16-treated group; c, *P* < 0.05 versus Tie2 KI + C16 group.

We measured MBP expression using Western blot analysis (**Figures 5D–F**) and IHC (**Figure 6**, red) in the different groups. The Tie2 KI + C16 and C16 peptide + LY294002-treated groups showed similar reduction in MBP expression at 3 weeks P.I., but treatment with C16 upregulated MBP expression comparable to the level in normal rats (**Figures 5D–F**). A similar trend was observed at 8 weeks P.I. for all groups, except for the observation that Tie2 KI + C16 treatment prevented significant downregulation of MBP expression to the extent observed at 3 weeks P.I.

We measured the expression of the neuronal marker NF-200 to assess the number of neurofilaments in the central and peripheral nervous system (**Figure 6**, green). Neurofilaments were significantly reduced in the SC of vehicle-treated NMO rats (**Figures 6B, F, J, K**). In contrast, treatment with C16 prevented demyelination and axonal loss (**Figures 6C, G, J, K**). Similarly, demyelination and axonal loss were prevented by Tie2 KI + C16 treatment (**Figures 6D, H, J, K**), but to a lesser degree compared to the C16-treated group. The C16 peptide + LY294002-treated group (**Figures 6E, I–K**) showed significant demyelination and axonal loss similar to that observed for the vehicle-treated group.

### C16 and Tie2 KI + C16 Reverse the Loss of APQ4 within the ON and SC and Increase GFAP Expression in the SC

**Figure 7:**
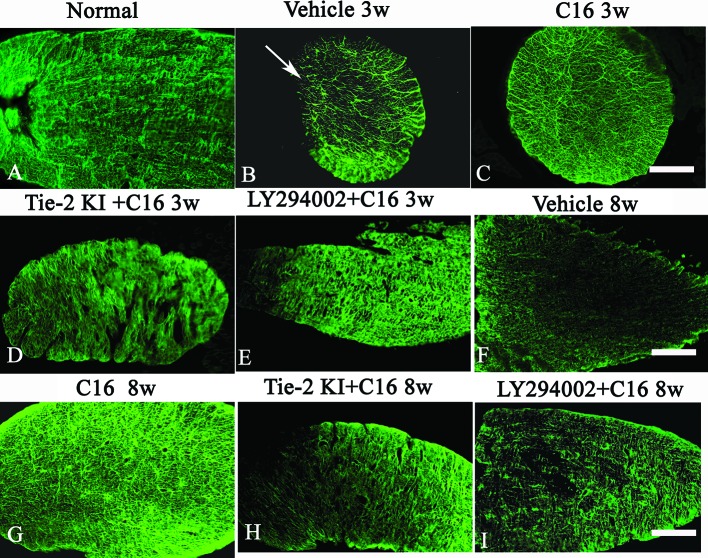
Aquaporin 4 (AQP4) immunostaining of the transverse sections through the optic nerve (ON) of normal **(A)** and neuromyelitis optica (NMO) rats treated with **(C, G)** C16, **(D, H)** Tie2 KI + C16, and **(E, I)** LY294002 + C16. *Scale bar*, 100 μm. AQP4 loss in the ON of vehicle-treated NMO rats is indicated by the *arrow* in **(B)**. **(F)** showed that expression of AQP4 was significantly reduced in the vehicle-treated NMO rats at 8 weeks post-immunization (pi).

**Figures 7A** is the normal group, **7B** and **7F** showed that expression of AQP4 was significantly reduced in the vehicle-treated NMO rats at both 3 (B) and 8 (F) weeks post-immunization (pi). **Figures 7C and G** showed that C16 treatment restored AQP4 to near normal levels at both 3 (C) and 8 (G) weeks pi. We observed slightly higher AQP4 levels in the Tie2 KI + C16 group (**Figure 7D** 3w pi, **Figure 7H** 8w pi), while no significant difference in AQP4 expression was noted in the C16 + LY294002-treated group (**Figure 7E** 3w pi, 7 I 8w pi) compared to the vehicle treated group (**Figures 7B, F**) at both 3 and 8 weeks pi.

**Figure 8:**
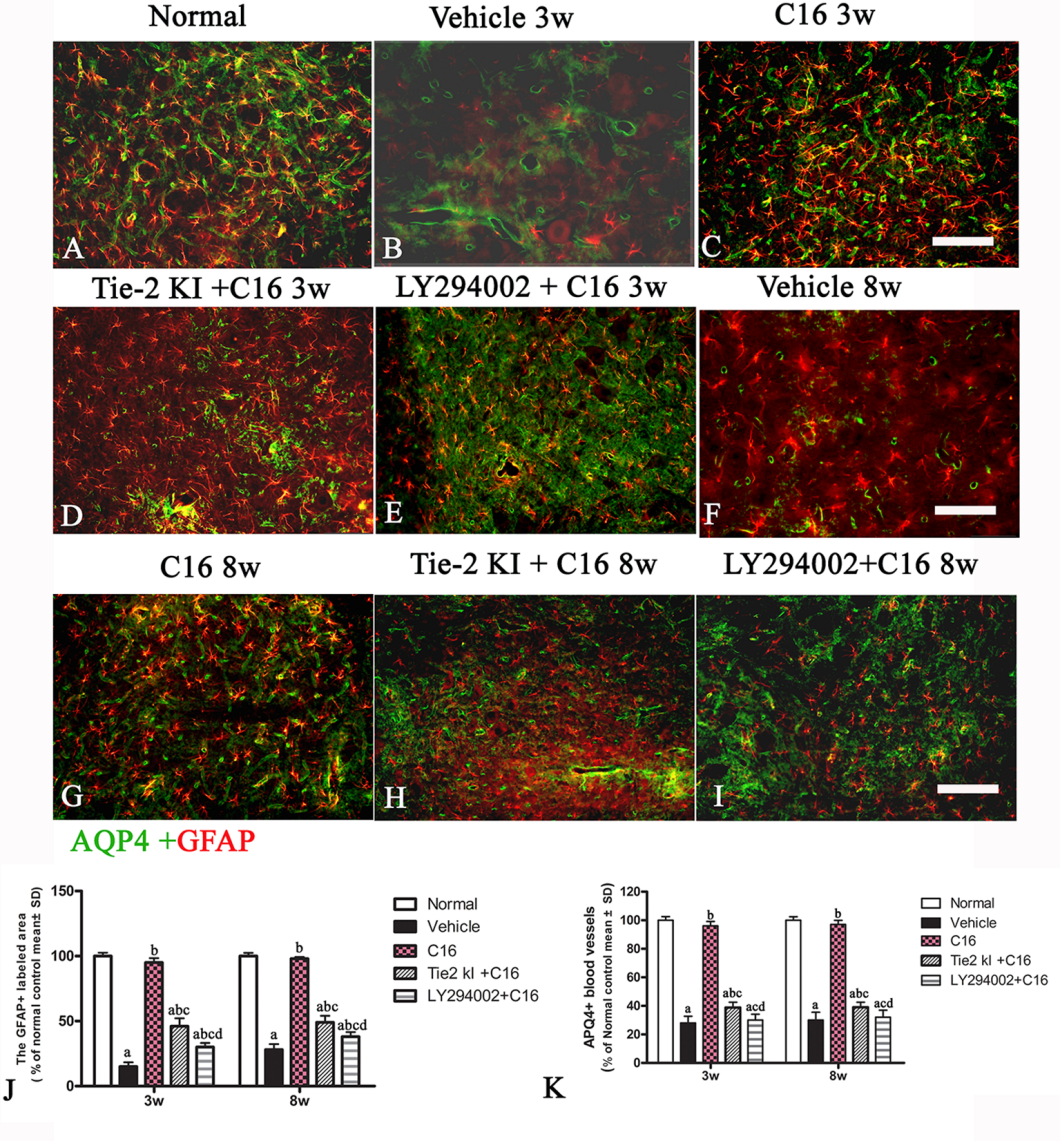
Double immunofluorescence staining images of the transverse sections through the anterior horn of the lumbar spinal cord (SC) showing the distribution of aquaporin 4 (AQP4, *green*) and glial fibrillary acidic protein (GFAP, *red*) in **(A)** normal and neuromyelitis optica (NMO) rats of the **(B, F)** vehicle-treated group, **(C, G)** C16 group, **(D, H)** Tie2 KI + C16 group, and **(E, I)** C16 peptide and LY294002-treated group at 3 and 8 weeks post-immunization (P.I.) (GFAP, *red*; AQP4, *green*). *Scale bar*, 100 μm. Plots of **(J)** GFAP^+^ cells and **(K)** APQ4^+^ blood vessels. a, *P* < 0.05 versus normal group; b, *P* < 0.05 versus the vehicle-treated group; c, *P* < 0.05 versus the C16-treated group; d, *P* < 0.05 versus Tie2 KI + C16 group.

**Figures 8A** is the normal group, the expression of specific astrocyte marker GFAP (**Figures 8B, F**) were significantly reduced in the vehicle-treated NMO rats at both 3 (8B) and 8 (8F) weeks pi. C16 treatment restored GFAP expression to near normal levels (**Figure 8A**) at both 3(C) and 8 (G) weeks pi. Slightly higher GFAP levels were found in the Tie2 KI + C16 group (**Figure 8D** 3w pi, 8H 8w pi), while no significant difference in GFAP expression was noted in the C16 peptide +LY294002-treated group (**Figure 8E** 3w pi, 8I 8w pi) compared to the vehicle treated group (**Figures 8B, F**) at both 3 and 8 weeks pi.

### C16 and Tie2 KI + C16 Protect Blood Vessel Integrity and Reduce Leakage

**Figure 9:**
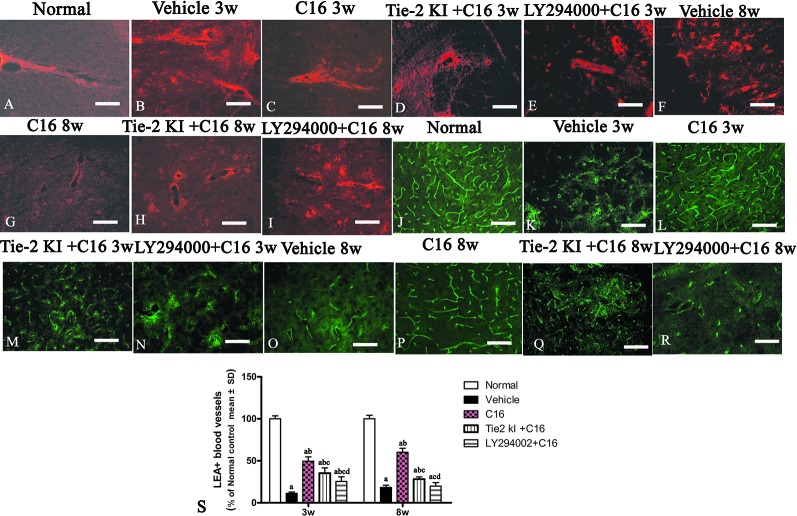
**(A–I)** Evans Blue (EB) staining (*red*) of the spinal cord (SC) of **(A)** normal and neuromyelitis optica (NMO) rats treated with **(B, F)** vehicle control, **(C, G)** C16, **(D, H)** Tie2 KI + C16, and **(E, I)** LY294002 + C16. **(J–R)** *L. esculentum* agglutinin lectin (LEA, *green*) injection to evaluate the extent of rescue of perfused blood vessels in the SC of **(J)** normal rats and NMO rats treated with **(K, O)** vehicle control, **(L, P)** C16, **(M, Q)** Tie2 KI + C16, and **(N, R)** LY294002 + C16. *Scale bar*, 100 μm. **(S)** LEA-injected blood vessels of NMO rats of the C16, Tie2 KI + C16, and LY294002 + C16 treatment groups. a, *P* < 0.05 versus normal group; b, *P* < 0.05 versus the vehicle-treated group; c, *P* < 0.05 versus the C16-treated group; d, *P* < 0.05 versus Tie2 KI + C16 group.

Our EB extravasation experiment revealed severe vasculature leakage in vehicle-treated rats (**Figures 9B, F**), when compared with the normal group (**Figure 9A**), but there was notably reduced leakage from surrounding blood vessels in the C16-treated group (**Figures 9C, G**). Similarly, reduced vascular leakage was observed in the Tie2 KI + C16 group (**Figures 9D, H**), but to a lesser degree compared to the C16-treated group. Visible blood vessel leakage similar to that of the vehicle-treated rats was observed in the C16 peptide + LY294002-treated group (**Figures 9E, I**).

**Figure 9S** is a plot of LEA-labeled blood vessels numbers in NMO rats of the C16, Tie2 KI + C16, and LY294002 + C16 treatment groups. The number of blood vessels in the SC of vehicle-treated rats was significantly reduced at 3 (**Figure 9K**) and 8 (**Figure 9O**) weeks P.I. compared to the normal control (**Figure 9J**). LEA injection restored blood vessel integrity to different degrees at both 3 and 8 weeks P.I. with the most significant improvement observed in the C16-treated group (**Figures 9L, P**), moderate improvement in the Tie2 KI + C16 group (**Figures 9M, Q**), and no significant improvement in the C16 peptide and LY294002-treated group (**Figures 9N, R**).

### C16 Treatment Prevents Infiltration of Inflammatory Cells, Edema, Demyelination, and Axonal and Neuronal Loss

**Figure 10:**
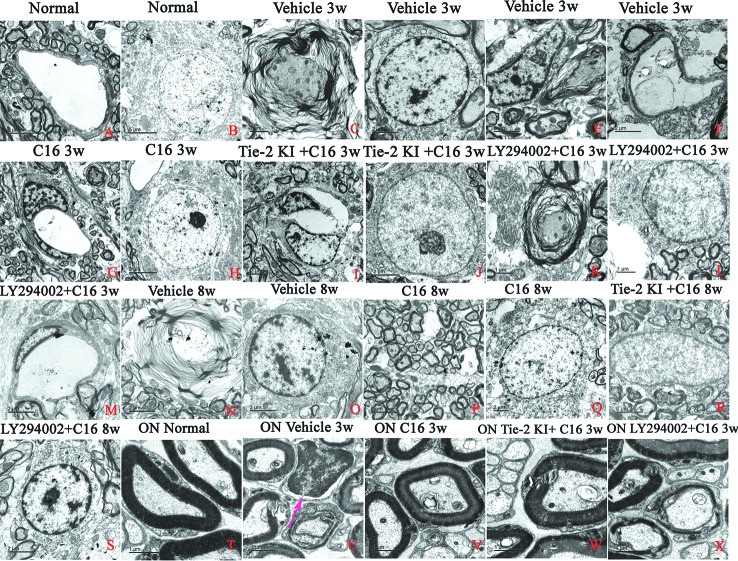
TEM micrographs of the spinal cord (SC) white matter, and optic nerve (ON) in **(A, B, T)** normal and **(C–S, U–X)** neuromyelitis optica (NMO) rats of the four treatment groups (vehicle, C16, Tie2 KI + C16, and LY294002 + C16) at 3 and 8 weeks post-immunization (P.I.) to evaluate the degree of perivascular edema, demyelination, axonal loss, and neuronal apoptosis. Arrow in **(U)** indicates infiltration of inflammatory cells within the ON. *Scale bar*: 2 μm in **(A, D–G, I**, **J, M–S)**; 1 μm in **(C, K, L, T–X)**; and 5 μm in **(B, H)**.

Unlike control rats, which showed normal nuclei with uncondensed chromatin (**Figures 10A, B**), TEM images of the SC and white matter of vehicle-treated NMO rats at 3 weeks P.I. revealed myelin sheath splitting and vacuolar changes (**Figure 10C**), apoptotic neurons with shrunken axons showing condensed, fragmented, and margination of nuclear chromatin (**Figure 10D**), infiltration of inflammatory cells within the SC tissue (**Figures 10E, U**), and severe blood vessel leakage and tissue edema in the extracellular space surrounding the vessels (**Figure 10F**) at 3 weeks P.I. Similar observations were found in the C16 peptide and LY294002-treated group at 3 weeks P.I. for myelin splitting (**Figure 10K**), apoptotic changes in the nuclei (**Figure 10L**), and perivascular edema (**Figure 10M**). Fewer vacuolated myelin sheaths, less severe perivascular edema (**Figures 10G, I**), and nuclei with normal ultrastructures (**Figures 10H, J, V, W**) were observed in both the ON and white matter of the SC in the C16 and Tie2 + C16 groups at 3 weeks P.I. At 8 weeks P.I., notable demyelination, severe blood vessel leakage and tissue edema, and signs of apoptosis (**Figures 10N, O**) were detected in the vehicle-treated group, but myelinated fibers were relatively intact in the C16 and Tie2 + C16 groups (**Figures 10P–R**) and their neighboring nuclei showed normal ultrastructures (**Figures 10Q, R**). Apoptotic changes in the nuclei were still apparent in the C16 peptide and LY294002-treated group (**Figure 10S**).

Within the ON (**Figures 10T–X**), vacuolated and fused myelin sheaths (**Figures 10U, X**) and infiltration of inflammatory cells (**Figure 10U**) were observed in the vehicle-treated and C16 peptide + LY294002-treated groups, while most of the normal structures were observed in the C16 and Tie2 + C16 groups (**Figures 10V, W**).

### C16 and Tie2 KI + C16 Suppress Expression of Pro-Inflammatory Factors IL-1, IL-8, and TNF-α, But Promote Expression of Anti-Inflammatory Cytokine IL-10

**Figure 11:**
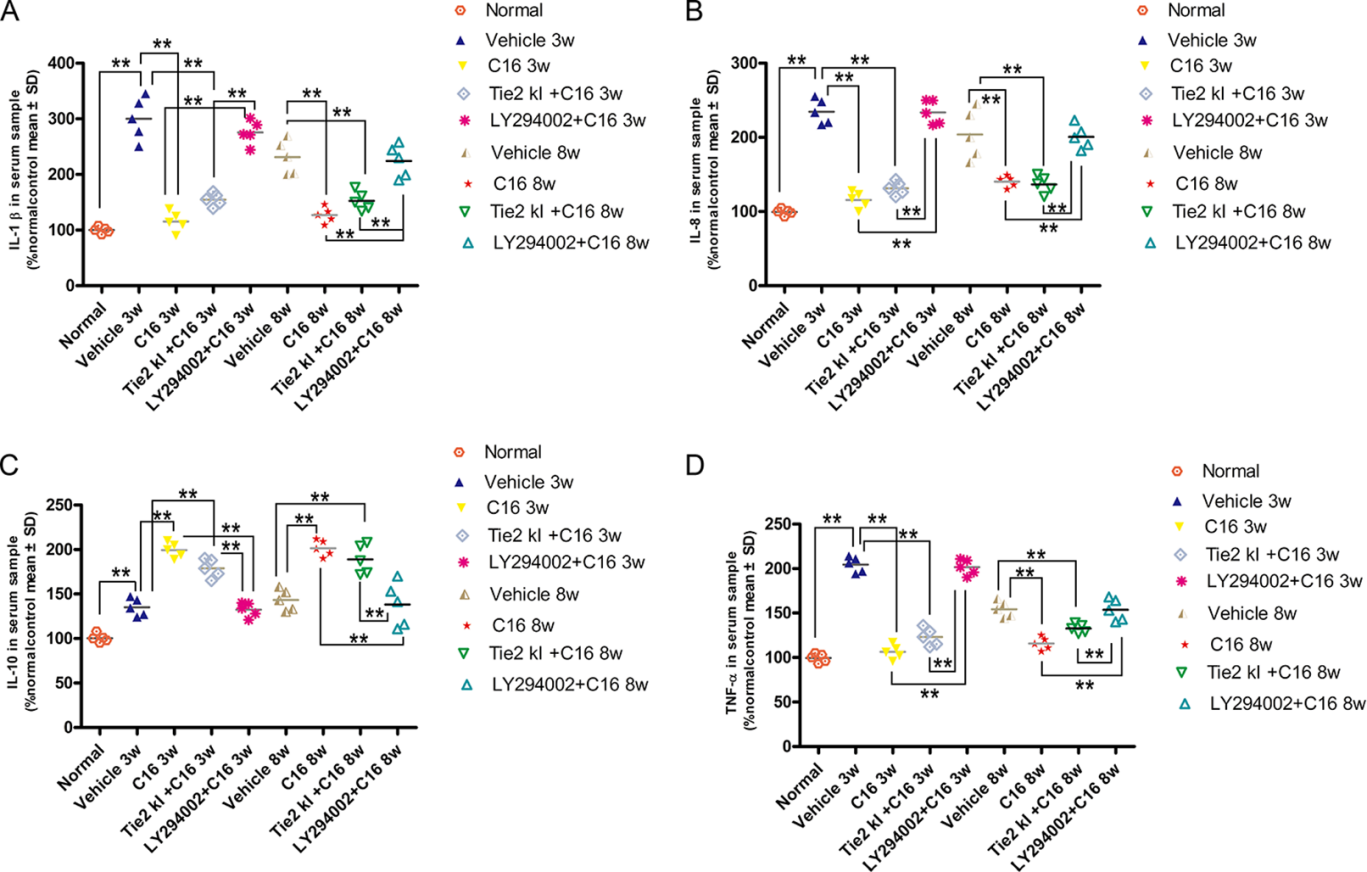
ELISA results of the expression levels of the pro-inflammatory cytokines **(A)** IL-1, **(B)** IL-8, and TNF-α **(D)** and anti-inflammatory cytokine IL-10 **(C)**. *n* = 5 in each group. **P < 0.01.

Expression levels of the pro-inflammatory cytokines TNF-α, IL-1β, and IL-8 were significantly increased in vehicle-treated NMO rats, while expression of these markers was significantly reduced in the C16 and Tie2 KI + C16, but not the LY294002 + C16, groups (**Figures 11A, B, D**). In contrast, IL-10 was downregulated in vehicle-treated NMO rats compared to normal rats at 3 and 8 weeks P.I. Treatment with C16 or Tie2 KI + C16, but not with LY294002 + C16, reversed these effects (**Figure 11C**).

### Tie2 KI and LY294002 Inhibitors Prevent Tie2 and Akt Kinase Expression

**Figure 5U** showed that the expression of pAKT was decreased in NMO model at 8 weeks pi while the C16 application could reverse this phenomenon, and the inhibition effects of pAkt expression by Tie2 and LY294002-specific inhibitors at 8 weeks pi. Inhibition of Tie2 and Akt/pAkt by Tie2 and LY294002-specific inhibitors was confirmed using Western blot analysis (Figures 5M–T) and immunofluorescence staining (Figures SP1 and SP2).

### C16, Tie2 KI + C16, and LY294002 + C16 Treatment Restores αVβ3- and αVβ1-Integrin Expression

Western blot analysis (**Figures 5V–Y-a**) and immunofluorescence staining (αvβ3, green; **Figures SP3A–J**; αvβ1, red; **Figures SP3K–T** revealed decreased neuronal expression of αvβ3 and α5β1 integrins due to NMO-IgG infusion in vehicle-treated NMO rats. The decrease in αvβ3 and α5β1 integrin expression was reversed in the C16, Tie2 KI, showed by Plots of αvβ3 and α5β1 positive neurons in anterior horn of spinal cord (**Figure SP3 U-V**), and the western blotting analysis (**Figure 5V–Y-a**). In **Figure 5**, Panel Z is a plot of the western blotting results of α5β1 integrin expression in each group, which showed that the decrease in α5β1 integrin expression could be reversed by the C16, Tie2 KI + C16, and LY294002 + C16 treatment.

## Discussion

We previously showed that C16 inhibited the trans-endothelial migration of C8166-CD4 lymphoblast cells and significantly reduced the infiltration of leukocytes and macrophages in the SC and brain in an experimental autoimmune encephalomyelitis (EAE) model, which is a frequently used MS animal model (Zhang et al., 2014; Wang et al., 2016; Tian et al., 2017). EAE animal models can help in identifying and elucidating several aspects of MS biology, including inflammation, immune surveillance, and immune-mediated tissue injury. In the EAE model, infiltration of circulating leukocytes into the CNS induces inflammation and myelin/axonal damage (Fang et al., 2013). The acute EAE model induced in Lewis rats, *via* subcutaneous injection of a mixture of guinea pig spinal cord homogenate (GPSCH) and complete Freud adjuvant (CFA), is characterized by a single episode of paralysis followed by spontaneous recovery. Thus, this enables the elucidation of the time points corresponding to the induction, peak, and resolution of the inflammation-based immune response during MS. In acute EAE models, the induced animals rapidly exhibit significant leukocyte infiltration throughout their brains and spinal cords after EAE onset (Fang et al., 2013). The activated microglia may induce inflammation, demyelination, myelin phagocytosis *via* receptor-mediated pathways, and disrupt BBB integrity. Therefore, targeting the neuroinflammatory reaction has been the common approach to protect motor neurons and alleviate clinical motor symptoms.

MOG35-55-immunized C57BL/6 mice develop a chronic non-remitting paralytic EAE disease (Zhang et al., 2014) that is reportedly associated with perivascular extravasation of CD45^+^ leukocytes. CD45^+^ leukocyte extravasation is an ongoing progressive inflammatory process that can remit several times and cannot recover spontaneously (Zhang et al., 2014). The early influx of macrophages and CD68-labeled active microglia were thought to sustain the pathological process. In our previous studies, C16 peptide application ameliorated the symptoms in both acute and chronic progressive EAE/MS models by reducing the neuroinflammatory responses (Fang et al., 2013; Han et al., 2013; Zhang et al., 2014).

The presence of NMO-IgG distinguishes the NMO model from MS. NMO-IgG selectively binds to AQP4. Pathological characteristics of NMO include loss of AQP4 and GFAP, granulocyte and macrophage infiltration, as well as demyelination and axonal injury primarily in the spinal cord and optic nerves (Zhang et al., 2018). The pathogenicity of NMO-IgG (anti-AQP4 autoantibodies) and human complement has been found to contribute to the initial NMO model (Zhang et al., 2018). The procedure for establishing the NMO model involves daily intraventricular and intrathecal delivery of (1 µl/h) AQP4-Ab and human complement for 3 days, unlike that for establishing the MS model. Both MS and EAE models can be used to study different aspects of inflammation, demyelination, re-myelination, and neurodegeneration in the central nervous system. However, pathogenesis such as optic nerve injury in the NMO model can often lead to blindness, which was rarely found in the EAE/MS model. Current EAE models are primarily based on inflammation that is induced by autoreactive CD4^+^ T-cells. Pathological data from previous studies reported the importance of CD8^+^ T-cells and B-lymphocytes in propagating inflammation and tissue damage in established MS model (Lassmann and Bradl, 2017). It is thus crucial to distinguish between NMO and MS because certain MS treatments could exacerbate NMO (Zhang et al., 2018). Given the similarities between NMO and MS, we speculated that C16 would exert comparable effects in the NMO model. Hence, we sought to elucidate the underlying mechanism of C16 peptide function in NMO.

Destruction of the BBB is the hallmark of NMO progression (Tasaki et al., 2014). Typically, NMO patients show loss of AQP4 and GFAP in SC lesions at the BBB, especially at active perivascular sites, as well as immune cell infiltration and loss of astrocytes. NMO is characterized by the selective binding of NMO-IgG to AQP4 at astrocytic foot processes at the BBB, thus destroying BBB integrity. This results in perivascular/parenchymal infiltration of leukocytes into the CNS. Thus, treatments that can maintain BBB integrity and/or prevent perivascular/parenchymal infiltration of inflammatory cells will be beneficial for NMO patients.

The synthetic peptide C16 (KAFDITYVRLKF) has a functional laminin domain that can selectively bind αvβ3 and α5β1 integrins. Previous studies showed that αvβ3 integrin could competitively inhibit monocyte transendothelial migration as well as regulate their migration from the vasculature to endothelia (Han et al., 2013; Zhang et al., 2018). Thus, C16 could competitively reduce the binding of inflammatory cells to the endothelium, thereby inhibiting leukocyte transmigration and relieving inflammatory immune responses in NMO patients. In the present study, we found that targeted prevention of the creation of a pro-inflammation microenvironment in the CNS of the NMO rat model could confer a protective effect.

In addition, ON damage in these patients results in RGC loss and myelin degeneration. Indeed, in the NMO rat model established in this study, we observed oligodendrocyte and neuronal apoptosis, decreased axon demyelination, inflammatory cell infiltration, and severe edema. We demonstrated that these pathological changes can be ameliorated by treatment with C16 peptide which delayed disease progression and severity. These findings are consistent with the results of our previous study using the EAE model (Zhang et al., 2018). The Tie2 KI and PI3K/Akt inhibitor LY294002 compromised the effects of C16, indicating the importance of the Tie2 and PI3K/Akt pathways in C16-mediated NMO therapy.

Of note, treating NMO rats with C16, Tie2 KI and C16, or LY294002 and C16 rescued αvβ3 and α5β1 integrin expression. Since Tie2 KI or LY294002 did not affect integrin expression, we conclude that C16 works upstream of Tie2 and PI3K/Akt signaling. Detectable Akt kinase expression at both 3 and 8 weeks P.I. despite inhibition of the Tie2-dependent pathway in the Tie2 KI-added C16 group suggests the existence of alternative pathways that support αvβ3 and/or α5β1 integrin-mediated PI3K/Akt activation, as well as increase the level of phosphorylated AKT (p-Akt). Possible alternative pathways include: the p53 pathway, which reportedly engages in a negative crosstalk with αvβ1 integrin (Renner et al., 2016); the α5β1-PI3K-Bcl-2 pathway, wherein α5β1 integrin induces p-Akt (Watabe et al., 2011); and the α5β1/αvβ3 integrin-dependent Akt, ERK, and JNK signaling pathways (Jin et al., 2011).

Although LY294002 and C16 treatment did not ameliorate the adverse pathological changes in NMO, GFAP expression was slightly restored and decrease in caspase-3 expression was prevented. This suggests that the PI3K/Akt signaling pathway is downstream of both Tie2 and Tie2 integrin pathways or directly downstream of αvβ1/αvβ3-dependent Akt signaling pathways. The minimal positive effects observed may indicate the involvement of the α5β1/αvβ3-dependent ERK and JNK pathways.

In conclusion, our findings indicate that the underlying mechanism of the C16 peptide involves three critical signaling pathways: Tie2-PI3K/Akt, Tie2–integrin, and integrin-PI3K/Akt, all of which promote vascular growth and stabilization and reduce inflammation in NMO.

## Data Availability Statement

The datasets generated and analyzed during the present study are available from the corresponding author upon reasonable request.

## Ethics Statement

The animal study was reviewed and approved by animal ethics committee of Zhejiang University.

## Author Contributions

Conception and design: SH. Financial support: HC. Administrative support: SH. Provision of study material or patients: HC. Collection and/or assembly of data: XF and JJ. Data analysis and interpretation: XF and JJ. Manuscript writing: SH and HC. All authors read and approved the final manuscript.

## Funding

We would like to acknowledge funding support from the Science and Technology Planning Project of Jinhua City, P.R. China (2018-4-060), Project of Enterprise Technology Research Consultant (grant no. 519000-I41701), Project of Enterprise Technology Research Consultant (grant no. 519000-I41801) and the National Natural Science Foundation of China, project no. 81971069.

## Conflict of Interest

The authors declare that the research was conducted in the absence of any commercial or financial relationships that could be construed as a potential conflict of interest.
