# Corrigendum: C16 peptide promotes vascular growth and reduces inflammation in a neuromyelitis optica model

**DOI:** 10.3389/fphar.2025.1611115

**Published:** 2025-05-13

**Authors:** Haohao Chen, Xiaoxiao Fu, Jinzhan Jiang, Shu Han

**Affiliations:** ^1^ Medical Molecular Biology Laboratory, School of Medicine, Jinhua Polytechnic, Jinhua, China; ^2^ Institute of Anatomy and Cell Biology, Medical College, Zhejiang University, Hangzhou, China

**Keywords:** C16 peptide, receptor tyrosine kinase Tie2, PI3K/Akt pathways, αvβ3 integrin, neuromyelitis optica (NMO)

In the published article, there was an error in **Supplementary Figure S1** as published. The magnification of subfigure B and G of **Supplementary Figure S1** is different from others. The corrected **Supplementary Figure S1** and its caption “Immunofluorescence images of Tie2 expression in transverse sections through the anterior horn of the lumbar SC in (A- I) normal and NMO rats treated with (C, G) C16, (D, H) Tie2 KI + C16 and (E, I) LY294002 + C16 at 3 and 8 weeks P.I. Scale bar = 100 μm. (J) Plot of Tie2+ blood vessels. a, *P* < 0.05 versus normal group; b, *P* < 0.05 versus the vehicle-treated group; c, *P* < 0.05 versus the C16-treated group; d, *P* < 0.05 versus Tie2 KI + C16 group. Tie2 loss in the ON of vehicle-treated NMO rats is indicated by the arrow in B, F.” has been updated in the original article.

The authors apologize for this error and state that this does not change the scientific conclusions of the article in any way. The original article has been updated.

