# Supplementary material for: C16 Peptide Promotes Vascular Growth and Reduces Inflammation in a Neuromyelitis Optica Model
Source: Front Pharmacol. 2019 Dec 3;10:1373. doi: 10.3389/fphar.2019.01373 (PMC6902286; doi:10.3389/fphar.2019.01373)
Supplement: Supplementary file 1 [file DataSheet_1.pdf]

Supplementary Material

Figure SP1. Immunofluorescence images of Tie2 expression in transverse sections through the anterior horn of the lumbar SC in (A - I) normal and NMO rats treated with (C, G) C16, (D, H) Tie2 KI + C16 and (E, I) LY294002 + C16 at 3 and 8 weeks P.I. Scale bar = 100 μm. (J) Plot of Tie2+ blood vessels. a, $P < 0.05$ versus normal group; b, $P < 0.05$ versus the vehicle-treated group; c, $P < 0.05$ versus the C16-treated group; d, $P < 0.05$ versus Tie2 KI + C16 group. Tie2 loss in the ON of vehicle-treated NMO rats is indicated by the arrow in B, F.

Figure SP2. Immunofluorescence images of Akt expression in transverse sections through the anterior horn of the lumbar SC in (A) normal and NMO rats treated with (C, G) C16, (D, H) Tie2 KI + C16 and (E, I) LY294002 + C16 at 3 and 8 weeks P.I. Scale bar = 100 μm. (J) Plot of Akt+ neurons in the anterior horn of SC. a, $P < 0.05$ versus normal group; b, $P < 0.05$ versus the vehicle-treated group; c, $P < 0.05$ versus the C16-treated group; d, $P < 0.05$ versus Tie2 KI + C16 group. Akt loss in the ON of vehicle-treated NMO rats is indicated by the arrow in B, F.

Figure SP3. Immunofluorescence images of (A - J, green) αvβ3 and (K - T, red) α5β1 integrin expression in transverse sections through the anterior horn of the lumbar SC in normal and NMO rats at 3 and 8 weeks P.I. Treating NMO rats with C16, Tie2 KI + C16, or LY294002 + C16, rescued αvβ3 and α5β1 integrin expression. Scale bar = 100 μm. (U) Plot of αvβ3+ neurons in the anterior horn of the

SC. (V) Plot of α5β1+ neurons in the anterior horn of the SC. a, $P < 0.05$ versus normal group; b, $P < 0.05$ versus the vehicle-treated group; c, $P < 0.05$ versus the C16-treated group; d, $P < 0.05$ versus Tie2 KI + C16 group.

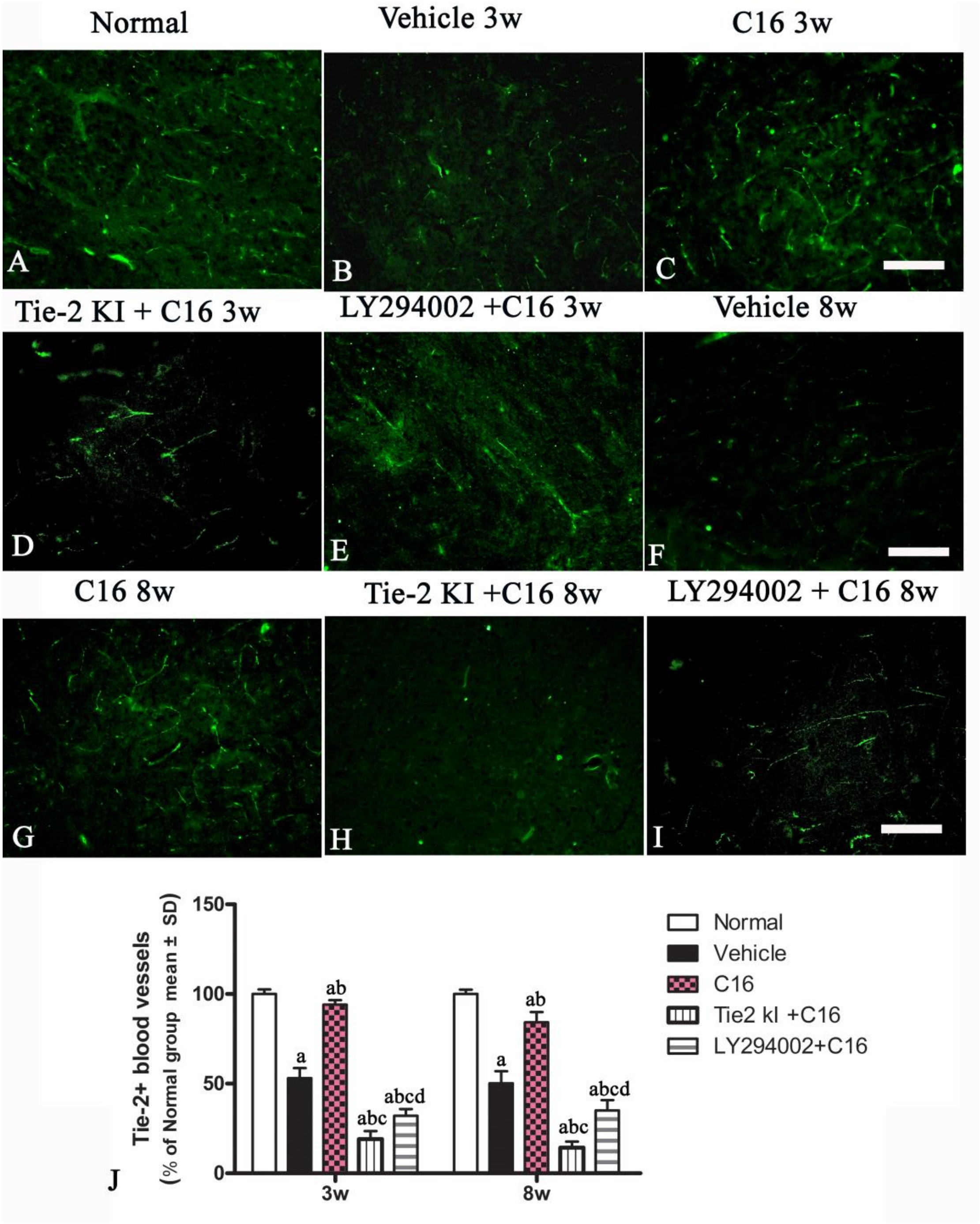
Normal
Vehicle 3w
C16 3w
A
B
C
Tie-2 KI + C16 3w
LY294002 +C16 3w
Vehicle 8w
D
E
F
C16 8w
Tie-2 KI +C16 8w
LY294002 + C16 8w
G
H
I
Tie-2+ blood vessels
(% of Normal group mean ± SD)
150
100
50
0
a
ab
abc
abcd
3w
8w
J
Normal
Vehicle
C16
Tie2 kI +C16
LY294002+C16

Figure SP1

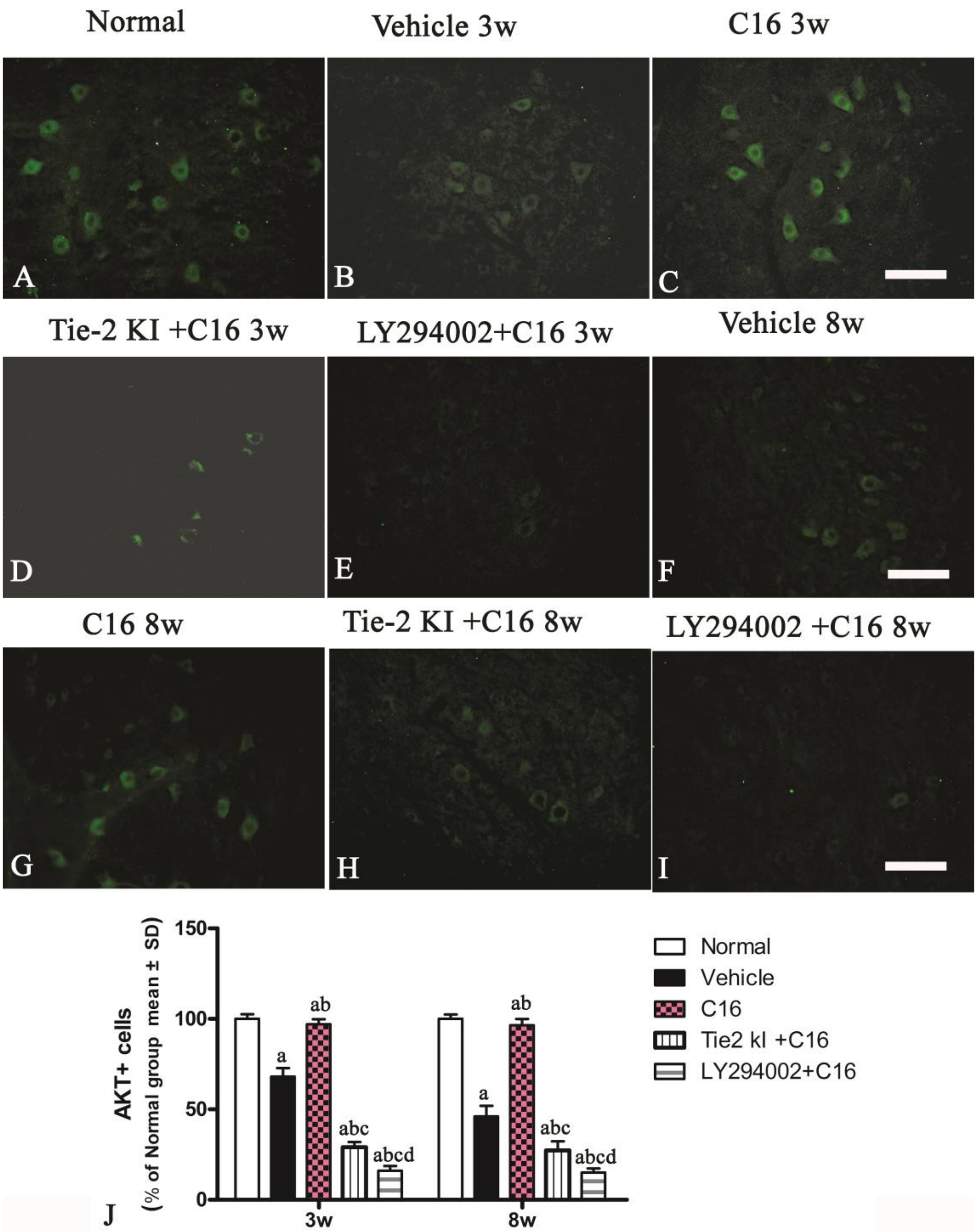
Normal
Vehicle 3w
C16 3w
A
B
C
Tie-2 KI +C16 3w
LY294002+C16 3w
Vehicle 8w
D
E
F
C16 8w
Tie-2 KI +C16 8w
LY294002 +C16 8w
G
H
I
AKT+ cells
(% of Normal group mean ± SD)
150
100
50
0
a
ab
abc
abcd
3w
8w
J
Normal
Vehicle
C16
Tie2 kI +C16
LY294002+C16

Figure SP2

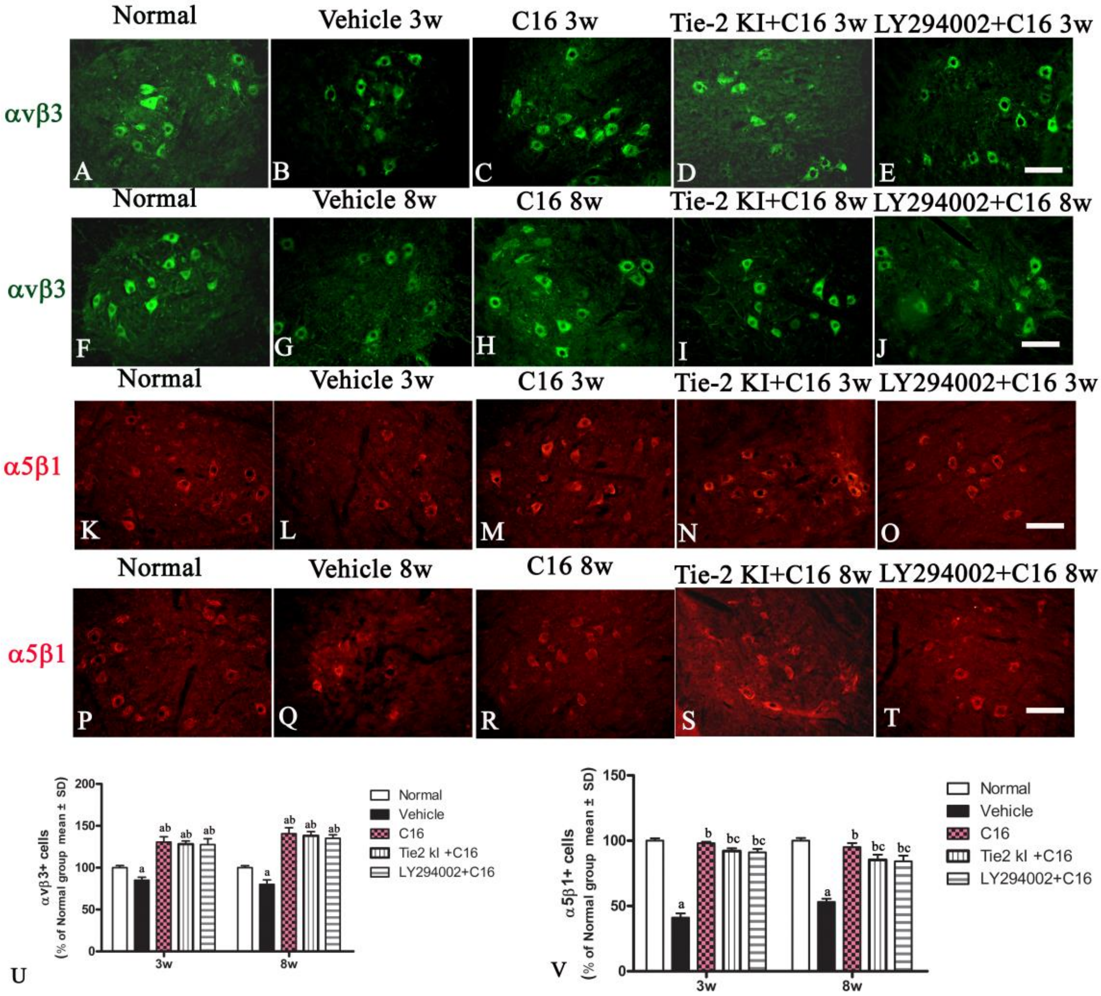

Normal
Vehicle 3w
C16 3w
Tie-2 KI+C16 3w
LY294002+C16 3w
αvβ3
A
B
C
D
E
Normal
Vehicle 8w
C16 8w
Tie-2 KI+C16 8w
LY294002+C16 8w
αvβ3
F
G
H
I
J
Normal
Vehicle 3w
C16 3w
Tie-2 KI+C16 3w
LY294002+C16 3w
α5β1
K
L
M
N
O
Normal
Vehicle 8w
C16 8w
Tie-2 KI+C16 8w
LY294002+C16 8w
α5β1
P
Q
R
S
T
αvβ3+ cells (% of Normal group mean ± SD)
Normal
Vehicle
C16
Tie2 kI +C16
LY294002+C16
3w
8w
U
α5β1+ cells (% of Normal group mean ± SD)
Normal
Vehicle
C16
Tie2 kI +C16
LY294002+C16
3w
8w
V

Figure SP3
